# Does the attention General Practitioners pay to their patients' mental health problems add to their workload? A cross sectional national survey

**DOI:** 10.1186/1471-2296-7-71

**Published:** 2006-12-05

**Authors:** Else M Zantinge, Peter FM Verhaak, Dinny H de Bakker, Klaas van der Meer, Jozien M Bensing

**Affiliations:** 1 NIVEL, Netherlands Institute for Health Services Research, P.O. Box 1568, 3500 BN Utrecht, The Netherlands; 2 Department of General Practice, University of Groningen, P.O. Box 196, 9700 AD Groningen, The Netherlands

## Abstract

**Background:**

The extra workload induced by patients with mental health problems may sometimes cause GPs to be reluctant to become involved in mental health care. It is known that dealing with patients' mental health problems is more time consuming in specific situations such as in consultations. But it is unclear if GPs who are more often involved in patients' mental health problems, have a higher workload than other GPs. Therefore we investigated the following: Is the attention GPs pay to their patients' mental health problems related to their subjective and objective workload?

**Methods:**

Secondary analyses were made using data from the Second Dutch National Survey of General Practice, a cross sectional study conducted in the Netherlands in 2000–2002. A nationally representative selection of 195 GPs from 104 general practices participated in this National Survey. Data from: 1) a GP questionnaire; 2) a detailed log of the GP's time use during a week and; 3) an electronic medical registration system, including all patients' contacts during a year, were used. Multiple regression analyses were conducted with the GP's workload as an outcome measure, and the GP's attention for mental health problems as a predictor. GP, patient, and practice characteristics were included in analyses as potential confounders.

**Results:**

Results show that GPs with a broader perception of their role towards mental health care do not have more working hours or patient contacts than GPs with a more limited perception of their role. Neither are they more exhausted or dissatisfied with the available time. Also the number of patient contacts in which a psychological or social diagnosis is made is not related to the GP's objective or subjective workload.

**Conclusion:**

The GP's attention for a patient's mental health problems is not related to their workload. The GP's extra workload when dealing in a consultation with patients' mental health problems, as is demonstrated in earlier research, is not automatically translated into a higher overall workload. This study does not confirm GPs' complaints that mental health care is one of the components of their job that consumes a lot of their time and energy. Several explanations for these results are discussed.

## Background

Mental health care is an important aspect of the General Practitioner's (GP's) job. GPs have a prominent position in signalling, and often also treating patients with mental health problems. But GPs are sometimes reluctant to become involved in their patient's mental health problems. They report that mental health care is one of the components of their job that places particular demands on their time and increases their perceived burden [1,2]. And the GP's workload is already an important topic because GPs often raise concerns about their increasing workload and lack of time with their patients [1,3].

A higher workload due to patients' mental health problems is, for example, expressed in longer consultations [4,7] and a higher contact rate for patients with mental health problems [5,6]. Furthermore, GPs more often experience a lack of time in consultations with patients with mental health problems [5], and more often feel stressed about these consultations [8]. These findings show that dealing with patients' mental health problems can be time consuming and demanding in specific situations such as in consultations. This we call a situational workload. We know that a GP's situational workload is higher in the case of mental health care. But it is not clear if the GP's extra situational workload, when dealing frequently with patients' mental health problems, is also translated into a higher workload overall. Therefore we looked at whether GPs who pay more attention to their patients' mental health problems have a higher workload than GPs who are more focussed on a patient's somatic problems.

It is well known that, regardless of the health care system and the patient population, the role that GPs play in mental health care, and their focus on mental health problems, varies widely [9,10]. Some GPs diagnose their patients' problems more often as psychological, while others are more inclined towards somatic interpretations. These differences in interpretation can be related to GPs' attitudes toward mental health problems, as some authors demonstrated [11,12]. While one GP will have a limited definition of mental health care tasks that they perceive as belonging to their role as a GP, others will perceive a broader spectrum of mental health care aspects as belonging to their tasks. However, it has not been known until now if differences in the GP's role perception and diagnosing of mental health problems also results in a variation in workload. Therefore we ask: Is the attention GPs pay to their patients' mental health problems related to their objective and subjective workload?

Our expectation is that paying more attention to a patient's mental health problems will result in more work for the GP. Patients with mental health problems contact their GP more frequently and their consultations take more time. Therefore, we expect that GPs with more patient contacts concerning patients' mental health problems will have more patient contacts in total and work more hours. Secondly, we expect that GPs who pay more attention to patients' mental health problems perceive their workload as higher, because GPs state that the patient's mental health problems are more demanding than other problems.

This paper describes the results of our study among general practitioners in order to answer the research question and test our expectations. We corrected for GP and patient characteristics that can affect the relationship between a GP's attention to mental health care and workload. GP characteristics that might affect the GP's workload are the GP's sex, age, working experience and personal list size [13-17]. It has also been shown that the degree of urbanisation of the practice and the kind of health insurance, sex, age, ethnicity, employment status and education level of the patients, might influence a GP's workload [8,13,17,18]. These characteristics are therefore included in the analyses.

## Methods

### Design

Secondary analyses were made using data from the Second Dutch National Survey of General Practice (DNSGP-2), a cross sectional study conducted in the Netherlands in 2000–2002 [19]. A nationally representative selection of 195 GPs from 104 general practices participated in this National Survey. Data were collected from general practitioners, other general practice personnel, and patients on the list of the participating practices. The Dutch National Survey sample is representative of the Dutch patient population, GPs and practices. The privacy of the participating persons is guaranteed in accordance with Dutch legislation [19].

An electronic medical registration of all patient contacts was used for our study. During a one-year period, 195 GPs in 104 practices kept an electronic record of all the contacts they had with their patients. The GP recorded the diagnoses of their patients, coded according to the International Classification of Primary Care (ICPC) [20]. Registration data from 96 out of the 104 practices were suitable for analysis. Eight practices were eliminated because their registration was incomplete. During one year, approximately 1.5 million contacts with patients were registered. Additionally, the participating GPs completed two written questionnaires, covering a wide range of topics, with response rates of 96% and 87% respectively. The GPs also kept a detailed log of their time use every quarter, by registering their activities during a representative working week. Here 84% responded. Patient characteristics were gathered from the practice registration and from a registration form that was sent to all patients on the lists of the participating practices. The response here was 77%.

### Measures

In table 1 an overview is provided of all measures used in this study and the type of data collection of the DNSGP-2 we applied.

**Table 1:** Overview of all measures used in this paper

**Measure**	**Type of data collection**
**Dependent**:	
*Workload*	
- Working hours weekly (objective)	GP diary 1 week
- Number of patient contacts weekly (objective)	Contact registration 1 year
- Satisfaction with the available time (subjective)	GP questionnaire
- Emotional exhaustion (subjective)	GP questionnaire
	
**Independent**:	
*GP's attention for mental health problems*	
- GP's role perception with respect to mental health problems	GP questionnaire
- % contacts with psychological or social diagnoses	Contact registration 1 year
	
*GP, practice and patient characteristics*	
- Sex, age, years of establishment, personal list size	GP questionnaire
- Degree of urbanisation	GP questionnaire
- % of publicly insured, women, 65+, non-Western, unemployed and low educated patients	Patient registration

### Objective workload (dependent)

The objective workload refers to the work that is done and the time that it takes. Two measures were used:

1) **Number of hours worked per week**. GPs registered in their diaries all the work activities they performed during a week. To prevent bias, the GPs were asked to register their time spent during a normal, representative working week.

2) The **number of patient contacts per week **was derived from the GPs' registration in their medical records during a year. The total number of patient contacts was divided by 52 to construct a measure of the mean number of contacts per week. A patient contact is an office consultation, telephone call or home visit.

### Subjective workload (dependent)

The subjective workload concerns the GP's perceived burden. Two indicators were used for the GP's subjective workload:

1) GP's **satisfaction with the time available**. In the GP questionnaire, GPs completed a job satisfaction scale, originally derived from McCranie (1982) [21]. According to a list of 16 working activities, the GPs recorded their satisfaction with that specific aspect of their job on a 5-point scale, ranging from 1 = very dissatisfied to 5 = very satisfied. Factor analysis showed a division into three sub-groups: satisfaction with the available time; satisfaction with the material aspects of the job; and satisfaction with colleague cooperation [17]. We made use of the sub-group 'satisfaction with the available time'.

2) GP's **emotional exhaustion**, one of the components of burnout. Burnout can be interpreted as a response to chronic stress; emotional exhaustion refers to feelings of energy depletion. In the GP questionnaire, the UBOS [22], a Dutch version of the Maslach Burnout Inventory [23], was used to measure levels of burnout. The UBOS-C consists of 20 items, ranging from 0 = never to 6 = always, that refer to feelings of emotional exhaustion, depersonalisation or reduced accomplishment. Mean scores are calculated for the exhaustion scale, taking into account the maximum allowed number of missing items [23].

### GPs' attention for mental health problems (independent)

Two indicators were used for the attention a GP pays to a patient's mental health problems:

1) **The GP's perception of his or her role in mental health care**. The GP questionnaire comprised a 5-point role perception scale about mental health care, originally derived from Grol [24]. According to a list of 10 mental health care activities, as for example 'discuss relationship problems' or 'support patients with addiction problems', the GP recorded if these activities belong to his or her tasks as a GP (1 = 'not' till 5 = 'fully'). Each GP's mean score on the role perception scale is then calculated.

2) **The percentage of patient contacts with a psychological or social diagnosis**. This information is derived from the contact registration of the DNSGP-2. We calculated, per GP, which part of all the recorded contacts during a year are contacts with at least one diagnosis in ICPC chapter P "Psychological" or Z "Social". We refer in this paper to these contacts as 'psychological contacts'. In the same contacts, somatic diagnoses may also have been made.

### Characteristics of GPs, patients and practices (potential confounders)

GP characteristics are derived from the GP questionnaire:

- Age, sex and years of establishment of the GP and FTE (Full Time Equivalent) hours worked

- Personal list size of the GP. The total number of patients on the practice list is distributed over the GPs in the practice according to their FTEs worked.

The practice and patient characteristics are derived from the GP questionnaire, and the patient registration of the DNSGP-2. In the Netherlands most GPs have fixed patient lists and every patient is registered with just one GP. However, in group practices patients are often able to visit GPs other than their own. In that case patients are sometimes only registered in a general practice, not for a specific GP. For this study the characteristics of the patients on the list of every GP were used when available (103 GPs). When the specific patient lists per GP were not available or not complete, characteristics from the practice population were used (88 GPs).

Adjustments were made in the analyses for the following characteristics:

- Degree of urbanisation of the practice (from 1-not urban to 5-very urban)

- % of publicly insured patients

- % of women patients

- % of patients older than 65 years

- % of patients of non-Western origin

- % of unemployed patients

- % of poorly educated patients (no, or only primary education)

### Analyses

The level of analysis is the GP. Analyses were performed using SPSS 11.5 software. First, descriptive statistics of the measures used in this paper are calculated. Several multiple regression analyses were conducted, with objective and subjective workload as outcome measures. The total explained variance is expressed in adjusted R squares. The influences of other GP, practice and patient variables are taken into account in the analysis. Because of the high correlation between a GP's age and years of experience (.91), the latter is excluded as a GP characteristic in the regression analyses. The percentage of unemployed patients and the percentage of poorly educated patients are also excluded, because of their high correlations with the percentage of patients of non-Western origin (.71), and the percentage of publicly insured patients (.67), respectively. The number of FTEs the GP works in practice is excluded due to the concurrence with personal list size.

## Results

In table 2, the descriptive statistics of the central variables of this paper are presented: the GP's workload; his or her attention to mental health problems; and GP, practice and patient characteristics. The variation between GPs is expressed in the variation coefficient (standard deviation/mean*100).

**Table 2:** Statistics describing workload, the GP's attention for mental health problems and GP, practice and patient characteristics

	**N**	**Mean (sd)**	**Variation coefficient**
*Workload*			
Hours worked weekly	154	43.72 (12.22)	27.95
Number of patient contacts weekly	133	112.64 (36.69)	32.57
Satisfaction with the available time (1–5)	164	2.91 (0.71)	24.40
Emotional exhaustion (0–6)	164	1.58 (0.79)	50.00
			
*GP's attention for mental health problems*			
Role perception (1'not'-5 'fully')	187	3.07 (0.50)	16.29
% of psychological contacts	141	9.34 (3.30)	35.33
			
*GP characteristics*			
% gender male	190	73%	-
Age	190	46.79 (6.58)	14.06
Personal list size	191	2072.30 (692.29)	33.41
			
*Practice and patient characteristics*			
Degree of urbanisation of practice (1–5)	190	3.01 (1.31)	43.52
% publicly insured patients	191	64.31 (9.14)	14.21
% female patients	191	50.55 (2.79)	5.52
% patients 65+	191	12.61 (4.96)	39.33
% non-Western patients	190	6.25 (11.27)	180.32

On average Dutch GPs work 44 hours a week. GPs are not very satisfied with their available time; a mean score of almost 3 means they are partly satisfied, partly dissatisfied. GPs' exhaustion scores are on average low: GPs report average scores between 1 and 2 on the burnout scales varying from 0 to 6. A score of 1 or 2 means that feelings of emotional exhaustion are found 'seldom' or 'sometimes'. With respect to the practice and patient characteristics, most variation between GPs is found in the percentage of patients of non-Western origin, while little variation is found in the percentage of women patients.

In table 3 the correlations between GPs' workload and their attention to mental health problems are presented. The objective workload measures are adjusted to the number of FTEs the GPs are working, in order to distinguish between busy and less busy GPs.

**Table 3:** Correlations between workload measures and the attention for mental health problems

	**1**.	**2**.	**3**.	**4**.	**5**.	**6**.
1. Working hours weekly/fte	1	-	-	-	-	-
2. Patient contacts weekly/fte	.03	1	-	-	-	-
3. Satisfaction time	-.15	.02	1	-	-	-
4. Emotional exhaustion	.01	.02	-42**	1	-	-
5. Role perception	.17*	-.04	.04	.10	1	-
6. % P or Z contacts	.16	-.02	.05	.08	.19*	1

*p < .05; ** P < .01

Table 3 shows that GPs with a broader perception of their role in mental health care work more hours a week. But on the other hand, GPs with a broader perception of their mental health care tasks do not have more patient contacts than GPs with a more limited role perception. The GP's percentage of contacts with a psychological or social diagnosis is also not significantly correlated to one of the objective workload measures. The GP's subjective workload is neither related to the GP's role perception, nor to his or her psychological contacts.

Table 3 also demonstrates that GPs' objective workload is not related to their subjective workload. GPs who work more hours weekly or have more patient contacts, are not more exhausted or unsatisfied with their available time compared to GPs with less working hours or patient contacts. GPs' working hours and their number of patient contacts, both objective workload measures, are also not correlated. The measures of subjective workload on the other hand are mutually related: GPs who are more satisfied with their available time are less exhausted. Additionally, the two measures that indicated GPs' attention to mental health problems are also correlated: GPs with a broader perception of their role in mental health care, more frequently reach a psychological or social diagnosis in the contacts with their patients.

Table 4 describes the results of a multiple regression analysis, to test the relationship between GPs' attention to mental health problems and their objective and subjective workload.

**Table 4:** Results of multiple linear regression analysis on the GP's objective and subjective workload, expressed in betas and explained variance (R^2^)

	**Working hours weekly (n = 120)**	**Patient contacts weekly (n = 127)**	**Job satisfaction time (n = 125)**	**Emotional exhaustion (n = 125)**
*GP's attention for mental health problems*				
Role perception	.04	-.09	.02	.09
% psychological contacts	-.03	.10	.09	.05
				
*GP characteristics*				
Gender male	.04	-.07	-.15	.07
Age	.17	.08	.17	-.19
Personal list size	.23*	.62**	-.08	-.03
				
*Practice characteristics*				
Degree of urbanisation	.13	-.19	-.14	-.05
% publicly insured patients	.14	-.04	-.19	.01
% female patients	.10	.12	-.17	.27*
% 65+ patients	-.13	-.05	.22*	-.16
% non-Western patients	-.02	.10	.18	-.07
				
R^2^	.06	.36**	.04	.07

*p < .05; **p < .01

Table 4 shows that neither GPs' perception of their role towards mental health problems, nor their percentage of psychological contacts, are related to GPs' objective and subjective workload. The number of patient contacts a GP deals with each week is the only workload measure significantly explained by the regression model. This significance can mainly be attributed to the strong relation with the GP's personal list size: GPs with a larger list size have more patient contacts. Additionally, table 4 shows that GPs with larger list sizes have longer working weeks. Two relationships are found between GPs' subjective workload and the practice population: The GP's satisfaction with the available time is associated with GPs with more older patients on their patient list and secondly, GPs are more exhausted when more women patients are on their patient lists.

## Discussion

Unexpectedly, GPs who pay more attention to their patients' mental health problems do not have a higher objective or subjective workload than GPs with less attention for mental health problems. Neither GPs' perception of their role towards mental health problems, nor their relative number of psychological or social diagnoses in patient contacts, are related to their workload. The GP's number of patient contacts is the only workload measure that is significantly explained by the GP, patient or practice characteristics. A strong positive relationship is found between the GP's number of patient contacts and the GP's personal list size. Bivariate analyses show that GPs with a broader perception of their role toward mental health care reach relatively more psychological or social diagnoses in the contacts with their patients than GPs with a narrower perception. Finally, we found that objective and subjective workload are substantially different concepts: no associations are found between GPs' objective workload and their feelings of dissatisfaction or exhaustion.

The finding that GPs' perceptions of their role towards mental health care is reflected in the diagnoses they make, agrees with earlier studies in which it is shown that GPs' attitudes toward mental health care affect their work [11,12]. This means that a doctor who wants to see a patient's mental health problems, will have a greater chance of finding them than GPs with a more limited perception of their role towards mental health problems.

As mentioned in the introduction, GPs' attention for patients' mental health problems may influence their workload in specific situations, such as in consultations where patients' psychological complaints play a part [4,7]. Our results show that this 'situational' workload on a specific day or moment is not translated into a higher workload overall. There are several possible explanations for the lack of relationship between GPs' attention for mental health problems and their workload. It is important to bear in mind that mechanisms can differ between GPs, and different processes can exist alongside each other.

One explanation is that a higher workload due to patients' psychological problems is compensated in other aspects of the GP's job. The GP's workload is affected by many factors that possibly override the influence of contacts with psychological diagnoses, as these contacts take, according to our results, only 9% of all GPs' patient contacts. Additionally, there is some decision room for GPs to get involved in the kind of problems and activities they prefer. GPs who feel comfortable with mental health problems, and who are competent in this field, probably spend some extra time and energy on their patients' mental health problems, but limit their involvement in other activities in which they feel less comfortable.

A second explanation is that the workload of GPs in itself also affects GPs' perception of their role in mental health care and their diagnoses of psychological problems, instead of the opposite relationship that we studied. Maybe GPs who have sufficient time available, who do not feel unduly stressed or burdened, are more likely to broaden their role in mental health care, show more openness towards their patients and make more psychological and social diagnoses. The same can be applied to GPs who have a lot of possibilities for referring patients, or for example, have support from a practice nurse. Conversely, when their workload is higher, or GPs' possibilities for referring patients or getting support are limited, GPs may compensate their higher workload by limiting their role in mental health care and by making fewer psychological and social diagnoses. This reasoning is supported by other authors who suggested that the workload itself may influence GPs' perception of their role [17,25]. or their focus on psychological aspects [26].

A last explanation is that patients suffering from mental health problems can be just as 'demanding' and time consuming for the GP irrespective of whether the GP designates their problems as psychological/social or as somatic. It is well known that a patient's mental health problems will not always be recognised and diagnosed by the GP [27,28]. GPs report that patients' psychological problems play a part in 20% of all consultations [2,29], while we found in this paper that a psychological diagnosis was made in 9% of all patient contacts. Some of the patients with mental health problems will get a psychological or social diagnosis while other mental health problems will remain unaddressed by the GP. And possibly assigning psychological or somatic diagnoses to this group of patients makes no difference with regard to a GP's workload. Probably diagnosing a patient's problems as psychological or social may even prevent excessive consulting. This explanation is supported by a trial of Roter (1995), who demonstrated that patients with mental distress who are recognised by their GP, visit their GP more often for a short period of time, but in the long run they do not visit their GP more often [30]. But one can argue if this arises from the recognition itself, or from the fact that the doctors who recognise more mental health problems deal with these problems more effectively compared to GPs who recognise them less.

### Limitations

One assumption made in this study is that GPs have fixed patient lists. The patient characteristics on the list of the GPs we controlled for in the regression analyses are based on the fictional situation that every patient visits only one GP. Although most patients in the Netherlands are registered with just one GP, patients in group practices may often be seen by other GPs. Patients can therefore self-select a GP dependent on their health problems. GPs with a broad perception of their role regarding mental health problems, are possibly visited more often by patients with mental health problems, resulting in more psychological diagnoses. This self-selection process cannot be adjusted for, due to the level of analysis (GP level instead of consultation level). It might partly explain the relationship between a GP's perception of his or her role and psychological diagnoses, but it cannot explain the lack of relationship between a GP's psychological diagnoses and his or her workload.

Secondly, the causes and effects of the studied relationships are unclear due to the cross sectional character of our study. It is not possible to determine what comes first: GPs' attention for their patients' mental health problems or GPs' workload.

## Conclusion

This study shows that the situational workload when dealing with patients' mental health problems, as demonstrated in other studies, is not automatically translated into a higher overall workload. GPs who pay more attention to their patients' mental health problems in their consultations are not more busy than other GPs, and they are as satisfied and exhausted as other GPs paying less attention to their patients' mental health problems. This study does therefore not confirm GPs' complaints that mental health care is one of the components of their job that consumes a lot of their time and energy.

## Abbreviations

GP – General Practitioner

DNSGP-2 – Second Dutch National Survey of General Practice

FTE – Full Time Equivalent

ICPC – International Classification of Primary Care

SD – Standard Deviation

## Competing interests

The author(s) declare that they have no competing interests.

## Authors' contributions

All authors read and approved the manuscript and gave final approval to the version to be published. EZ advanced the first conception of the study, performed the statistical analyses and drafted the manuscript. PV participated in the conception of the study and contributed especially to the intellectual content. DB participated in the conception of the study and assisted with the statistical analyses. KM and JB contributed especially to the interpretation and discussion of results.

## Pre-publication history

The pre-publication history for this paper can be accessed here:


